# Racial and Ethnic Disparities in Use of Helicopter Transport After Severe Trauma in the US

**DOI:** 10.1001/jamasurg.2024.6402

**Published:** 2025-01-22

**Authors:** Christian Mpody, Maíra I. Rudolph, Alexandra Bastien, Ibraheem M. Karaye, Tracey Straker, Felix Borngaesser, Matthias Eikermann, Olubukola O. Nafiu

**Affiliations:** 1 Department of Anesthesiology, Montefiore Medical Center and Albert Einstein College of Medicine, Bronx, New York; 2 Department of Anesthesiology, The Ohio State University, Columbus; 3 University of Cologne Faculty of Medicine, University Hospital Cologne, Department for Anesthesiology and Intensive Care Medicine, Cologne, Germany; 4 Department of Population Health, Hofstra University, Hempstead, New York; 5 Klinik für Anästhesiologie und Intensivmedizin, Universität Duisburg-Essen, Essen, Germany

## Abstract

**Importance:**

In the US, traumatic injuries are a leading cause of mortality across all age groups. Patients with severe trauma often require time-sensitive, specialized medical care to reduce mortality; air transport is associated with improved survival in many cases. However, it is unknown whether the provision of and access to air transport are influenced by factors extrinsic to medical needs, such as race or ethnicity.

**Objective:**

To examine the current trends of racial and ethnic disparities in air transport use for patients who sustain severe trauma.

**Design, Setting, and Participants:**

This population-based cohort study used data from the National Trauma Data Bank from 2016 to 2022. Participants were patients older than 15 years who sustained a severe injury and required an urgent surgical procedure or intensive care unit (ICU) admission at level I or II trauma centers with helicopter service.

**Exposure:**

Severe injury requiring treatment at a level I or II trauma center.

**Main Outcomes and Measures:**

The primary mode of transport, categorized as either helicopter ambulance or ground ambulance. A multifaceted approach was used to narrow the observed racial and ethnic disparities in helicopter deployment. The secondary outcome was mortality after helicopter transport vs ground ambulance transport.

**Results:**

Data were included for 341 286 patients at 458 level I or II trauma centers with helicopter service. Their mean (SD) age was 47 (20) years; 243 936 patients (71.6%) were male and 96 633 (28.4%) female. Asian individuals were less likely to receive helicopter transport compared with White individuals (6.8% vs 21.8%; aRR, 0.38; 95% CI, 0.30-0.48; *P* < .001), driven by lower use for Asian patients in teaching hospitals (aRR, 0.29; 95% CI, 0.21-0.40; *P* < .001) and level I trauma centers (aRR, 0.33; 95% CI, 0.24-0.44; *P* < .001). In addition, Black patients were less likely to receive helicopter transport (8.7% vs 21.8%; aRR, 0.42; 95% CI, 0.36-0.49; *P* < .001), particularly in teaching hospitals (aRR, 0.41; 95% CI, 0.33-0.50; *P* < .001) and level I trauma centers (aRR, 0.40; 95% CI, 0.34-0.49; *P* < .001). A similar but less pronounced disparity was noted for Hispanic patients. Helicopter transport was associated with a lower mortality risk compared with ground transport (37.7% vs 42.6%; adjusted relative risk [aRR], 0.87; 95% CI, 0.85-0.89; *P* < .001).

**Conclusion and Relevance:**

This study found that racial and ethnic minority patients, particularly Asian and Black patients, and notably those treated at level I teaching hospitals were less likely to receive airlift services compared with White patients. The current expansion of helicopter emergency medical services has yet to translate into equitable care for patients of all races and ethnicities.

## Key Points

**Question:**

Do racial and ethnic disparities exist in the use of air transport for patients with severe trauma in the US?

**Findings:**

In this population-based cohort study including data for more than 341 000 severely injured patients across 458 trauma centers, racial and ethnic minority patients, especially Asian and Black individuals, were significantly less likely to receive helicopter transport compared with White patients despite the associated survival benefits. These disparities were particularly pronounced in teaching hospitals and level I trauma centers.

**Meaning:**

The current expansion of air transport use after severe trauma in the US has yet to translate into equitable trauma care across racial and ethnic groups.

## Teaser

This cohort study examines data from the US National Trauma Data Bank for patients who required treatment at a level I or II trauma center with helicopter service to determine racial and ethnic disparities in use of air transport for patients with severe trauma.

## Introduction

Traumatic injuries are a leading cause of mortality in the United States, ranking within the top 5 across all age groups.^1^ In severe trauma, every second counts toward optimizing survival, an urgency encapsulated in the concept of the “golden hour.”^1^ This concept is based on the trimodal distribution of trauma-related survival, in which the first hour after the incident is crucial for delivering definitive care before the risks of morbidity and mortality increase dramatically.^2^ In this context, helicopter air ambulances emerge as a vital component of emergency medical services (EMS), reducing the time to definitive care and improving survival for severely injured patients.^3,4,5,6,7^

After the inception of helicopter transportation in the 1970s, coordinated EMS and helicopter use was able to dramatically improve trauma care.^8^ Current data show that more than a quarter of US residents (more than 80 million people) could only reach a level I or II trauma center within the golden hour if they were transported by helicopter,^9^ underscoring the central role of helicopter flight programs in trauma care and justifying their continued expansion. However, the scope of this expansion is not merely a matter of logistical efficiency; it must also account for another critical issue: equity.

Numerous studies,^10,11,12,13^ including our own,^14^ have documented inequity in trauma outcomes, with racial and ethnic minority patients bearing a disproportionate burden of mortality and morbidity. This inequity raises questions about whether nonclinical factors, such as race or ethnicity, inadvertently influence the deployment of life-saving interventions, such as air transport for severely injured patients. If such a disparity exists, its trends must be explored to catalyze corrective policy and system-level changes toward an equitable approach to emergency trauma care.

To address these knowledge gaps, we aimed to achieve 2 objectives. First, we sought to evaluate whether a racial or ethnic disparity exists in the risk-adjusted use of helicopter ambulances after severe trauma requiring urgent surgical procedures. Second, we aimed to evaluate whether the gaps in helicopter use between patients of different races and ethnicities have narrowed or widened over time.

## Methods

### Study Design and Dataset

We conducted a population-based study using data from the National Trauma Data Bank (NTDB), endorsed by the American College of Surgeons, between 2016 and 2022. Briefly, the NTDB is the largest trauma registry in the world, aggregating more than 6 million records collected from more than 900 US trauma centers.^15^ Participating hospitals report annual data in a process that uses a uniform set of data definitions. The NTDB includes a broad spectrum of data, including patient demographics, hospital demographics, general trauma assessments, hospital identifiers, physiology values, and in-hospital mortality. Further details about the NTDB have been published elsewhere.^15^ Because of the deidentified nature and use of publicly accessible data, this study did not require institutional review board approval or informed consent processes.

### Patients

**Figure 1.**
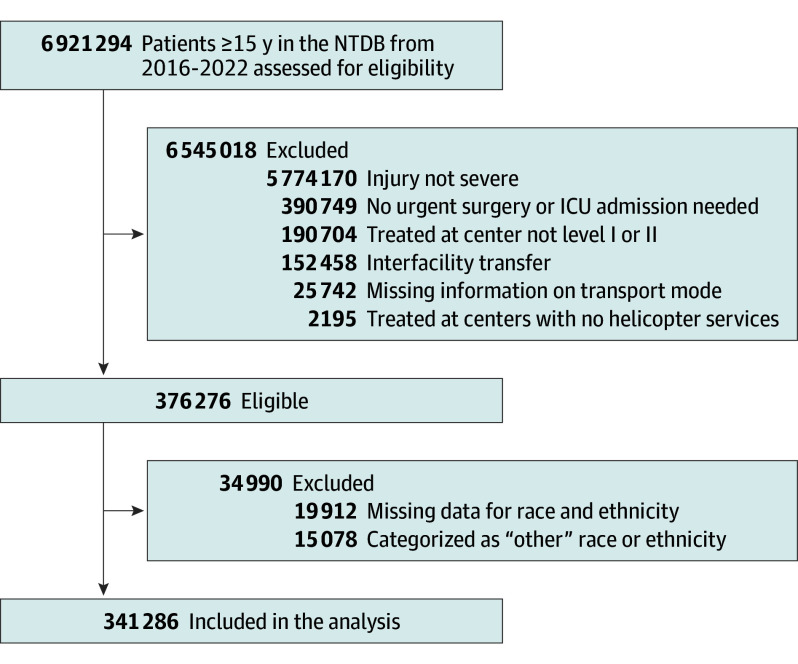
Flowchart ICU indicates intensive care unit; NTDB, National Trauma Data Bank.

We included all patients who sustained severe traumatic injuries and required urgent surgical procedures or intensive care unit (ICU) admission at level I or II trauma centers with helicopter service. As in previous research,^16^ severe injury was defined as an Injury Severity Score higher than 15, and urgent surgery was defined as emergency department disposition to the operating room. ICU admission was also defined based on the emergency department disposition. We only included patients who were transported by either helicopter or ground ambulance. We excluded patients who were transferred from another hospital and those whose race or ethnicity was unspecified (Figure 1).

### Race and Ethnicity

In the NTDB, race and ethnicity are self-reported by patients or provided by a family member and follow the categorization used by the US Census Bureau.^15^ In our study, race and ethnicity were categorized into 4 mutually exclusive groups: Asian, Hispanic, non-Hispanic Black, and non-Hispanic White (henceforth referred to as Asian, Hispanic, Black, and White, respectively).

### Outcomes

Our primary outcome was the transport mode (helicopter ambulance vs ground ambulance) used for different racial/ethnic groups. The secondary outcome was mortality following helicopter transport vs ground ambulance transport.

### Covariates

We adjusted our analysis for several patient and hospital characteristics that we selected based on the existing literature and our clinical judgment, which is also in agreement with recommendations on research using the NTDB^17^: age category (16-21, 22-34, 35-44, 45-54, 55-64, 65-74, 75-84, and ≥85 years), composite Injury Severity Score (continuous), sex (female, male), chronic complex condition (yes, no), injury type (blunt, penetrating, other), primary payer (private, public, other), intent of injury (unintentional, self-inflicted, assault, other), mechanism of injury (physical trauma, vehicle-related injuries, fall-related injuries, other), EMS-reported systolic blood pressure less than 90 mm Hg (yes, no), EMS-reported heart rate higher than 120/min (yes vs no), EMS-reported total Glasgow Coma Scale score less than 8 (yes, no), severe injury (Abbreviated Injury Scale score >3) in any region of the body (head/neck, face, chest, abdominopelvic, extremities), hospital size (≤200, 201-400, 401-600, >600 beds), hospital status (teaching, nonteaching, community), trauma center level (level I, level II), and hospital control (for-profit, nonprofit). In further analyses, we also adjusted for an indicator of the distance between the injury site and the receiving hospital among a subset of the study population with information on transport times (n = 200 948). To estimate the travel distance from scene to hospital, we use a previously used approach and applied average speeds of 20.1 mph for ground ambulances and 130 mph for helicopter ambulances based on previous research.^9,18^

### Statistical Analysis

We examined differences in baseline characteristics between patients of different races and ethnicities using the median (IQR) and frequency (percentage). We summarized the results of log-binomial regression models using the adjusted relative risk (aRR) values along with their 95% CIs, taking into account the clustering by trauma center. When the log binomial did not converge, Poisson regression modeling was used. To evaluate trends in racial and ethnic differences in helicopter ambulance use among the groups, we used log-binomial regression analysis with a 2-way interaction term between race and ethnicity category and year.^19^ Using this approach, we determined whether the disparity between racial and ethnic minority and White patients widened or narrowed over time while adjusting for baseline patient and hospital characteristics. We evaluated the extent of missing values and accommodated missing data by complete case analyses because the missingness was deemed small (<5%) and unlikely to alter the results. Because we performed multiple comparisons (2), we applied a Bonferroni correction and considered *P* values less than .025 (.05/2) statistically significant.

### Sensitivity Analyses

To evaluate the strength of our study’s results, we conducted multiple sensitivity analyses. Further details are included in the eMethods and eFigures 1-7 in Supplement 1. First, we repeated the analysis to account for the distance between the injury site and the receiving hospital among a subset of the study population with information on transport times (59% of the study population).^18^ Second, we repeated the analysis by conducting 1:1 propensity score matching sequentially to account for clinical characteristics that could influence the decision to use helicopter transport. Third, we repeated our primary analysis in subgroups categorized as either private or public insurance, as predefined within the NTDB.^20^ Fourth, we repeated the analyses for patients with similar injuries, specifically across different mechanisms of injury (blunt, penetrating) and levels of injury severity (severe, very severe). Fifth, to account for the differences between prehospital emergency services in urban and rural areas, where means of transportation might differ, we repeated our primary analysis in a subset of patients with a distance between site of injury and targeted hospital of more than 15 miles. Sixth, we evaluated the potential impact of unmeasured confounders by estimating the E-value.^21^ All analyses were performed using Stata version 16 (StataCorp).

## Results

### Characteristics of Patients

We identified 341 286 patients who sustained severe injuries requiring urgent surgery or ICU admission at 458 level I or II trauma centers with helicopter service between 2016 and 2022. Their mean (SD) age was 47 (20) years; 243 936 patients (71.6%) were male and 96 633 (28.4%) female (Table). Racial and ethnic minority patients were generally younger than White patients, with a higher proportion of Black and Hispanic patients in the age group 22 to 34 years. Additionally, Black patients were more likely to sustain penetrating injuries and assaults, while they and Hispanic patients were less likely to be female or have chronic conditions compared with White patients; they also more frequently received care at teaching hospitals and level I trauma centers. Notably, helicopter transport was associated with a lower mortality risk compared with ground transport (37.7% vs 42.6% for ground transport; aRR, 0.87; 95% CI, 0.84 to 0.90; *P* < .001) (eFigure 3 in Supplement 1).

**Table.** Characteristics of Patients With Severe Traumatic Injuries Who Required an Urgent Surgical Procedure or ICU Admission: NTDB 2016-2022^a^

Characteristic	No. (%)				
	Overall	Asian	Black	Hispanic	White
Study population	341 286 (100.0)	10 166 (3.0)	63 346 (18.0)	56 200 (16.5)	211 574 (62.0)
Age category, y					
16-21	33 627 (9.9)	620 (6.1)	8935 (14.1)	8030 (14.3)	16 042 (7.6)
22-34	79 642 (23.3)	1548 (15.2)	21 861 (34.5)	17 668 (31.4)	38 565 (18.2)
35-44	45 625 (13.4)	1017 (10)	10 214 (16.1)	9308 (16.6)	25 086 (11.9)
45-54	43 560 (12.8)	1115 (11)	7713 (12.2)	7272 (12.9)	27 460 (13)
55-64	48 383 (14.2)	1404 (13.8)	7481 (11.8)	5895 (10.5)	33 603 (15.9)
65-74	38 589 (11.3)	1600 (15.7)	4021 (6.3)	3692 (6.6)	29 276 (13.8)
75-84	31 019 (9.1)	1684 (16.6)	1860 (2.9)	2577 (4.6)	24 898 (11.8)
≥85	20 841 (6.1)	1178 (11.6)	1261 (2)	1758 (3.1)	16 644 (7.9)
Sex^b^					
Female	96 633 (28.4)	3859 (38.0)	14 056 (22.3)	12 329 (22.0)	66 389 (31.4)
Male	243 936 (71.6)	6294 (62.0)	49 062 (77.7)	43 757 (78.0)	144 823 (68.6)
Composite ISS, median (IQR)	24 (18-29)	24 (18-29)	25 (18-29)	24 (17-29)	24 (17-29)
Chronic complex condition	136 599 (40.0)	4726 (46.5)	19 123 (30.2)	14 622 (26)	98 128 (46.4)
Injury type					
Blunt	286 694 (84.0)	9220 (90.7)	40 438 (63.8)	45 705 (81.3)	191 331 (90.4)
Penetrating	43 401 (12.7)	544 (5.4)	21 025 (33.2)	8169 (14.5)	13 663 (6.5)
Other	11 191 (3.3)	402 (4.0)	1883 (3.0)	2326 (4.1)	6580 (3.1)
Primary payer					
Private	174 996 (52.4)	4937 (49.3)	31 093 (50.6)	29 436 (53.0)	109 530 (52.9)
Public	151 009 (45.2)	4840 (48.3)	28 699 (46.7)	24 087 (43.4)	93 383 (45.1)
Other	8107 (2.4)	236 (2.4)	1628 (2.7)	2007 (3.6)	4236 (2.0)
Intent					
Unintentional	280 855 (84.5)	8928 (90.9)	39 069 (63.6)	44 270 (81.5)	188 588 (91.2)
Self-inflicted	10 129 (3.0)	314 (3.2)	1319 (2.1)	1384 (2.5)	7112 (3.4)
Assault	40 490 (12.2)	565 (5.8)	20 845 (33.9)	8482,(15.6)	10 598 (5.1)
Other	972 (0.3)	10 (0.1)	240 (0.4)	191 (0.4)	531 (0.3)
Mechanism of injury					
Physical trauma	54 332 (15.9)	853 (8.4)	23 145 (36.5)	10 417 (18.5)	19 917 (9.4)
Vehicle related	181 021 (53.0)	4698 (46.2)	29 890 (47.2)	30 569 (54.4)	115 864 (54.8)
Fall related	90 455 (26.5)	4069 (40.0)	7940 (12.5)	12 041 (21.4)	66 405 (31.4)
Other	15 478 (4.5)	546 (5.4)	2371 (3.7)	3173 (5.6)	9388 (4.4)
EMS-reported vital signs					
SBP <90 mm Hg	39 821 (11.7)	875 (8.6)	9427 (14.9)	6698 (11.9)	22 821 (10.8)
Heart rate >120/min	55 229 (16.2)	1232 (12.1)	11 927 (18.8)	10 668 (19.0)	31 402 (14.8)
Total CGS score <8	93 152 (27.3)	2558 (25.2)	17 649 (27.9)	16 207 (28.8)	56 738 (26.8)
AIS location					
Head/neck	212 667 (62.3)	7263 (71.4)	32 814 (51.8)	33 962 (60.4)	138 628 (65.5)
Face	75 589 (22.1)	1811 (17.8)	12 851 (20.3)	13 296 (23.7)	47 631 (22.5)
Chest	163 200 (47.8)	3840 (37.8)	31 098 (49.1)	27 715 (49.3)	100 547 (47.5)
Abdominopelvic	140 131 (41.1)	3473 (34.2)	30 347 (47.9)	25 233 (44.9)	81 078 (38.3)
Hospital size in beds					
≤200	19 495 (5.7)	704 (6.9)	3402 (5.4)	3071 (5.5)	12 318 (5.8)
201-400	86 448 (25.3)	2882 (28.3)	11 255 (17.8)	14 266 (25.4)	58 045 (27.4)
401-600	102 338 (30.0)	3627 (35.7)	17 311 (27.3)	22 169 (39.4)	59 231 (28.0)
>600	133 005 (39.0)	2953 (29.0)	31 378 (49.5)	16 694 (29.7)	81 980 (38.7)
Hospital teaching status					
Teaching	187 310 (55.2)	5636 (55.5)	42 293 (67.1)	31 917 (57.0)	107 464 (51.1)
Nonteaching	152 191 (44.8)	4519 (44.5)	20 691 (32.9)	24 093 (43.0)	102 888 (48.9)
Trauma center level					
Level I	227 336 (66.6)	6618 (65.1)	49 716 (78.5)	36 885 (65.6)	134 117 (63.4)
Level II	113 950 (33.4)	3548 (34.9)	13 630 (21.5)	19 315 (34.4)	77 457 (36.6)

Abbreviations: AIS, Abbreviated Injury Scale; EMS, emergency medical services; GCS, Glasgow Coma Scale; ICU, intensive care unit; ISS, injury severity score; NTDB, National Trauma Data Bank; SBP, systolic blood pressure.

^a^ We retained data for 341 286 patients (age >15 years) who sustained a severe injury and required an urgent surgical procedure or ICU admission at a level I or II trauma center reporting to the NTDB.

^b^ Sex data were missing for 717 cases.

### Transport Mode by Race and Ethnicity

**Figure 2.**
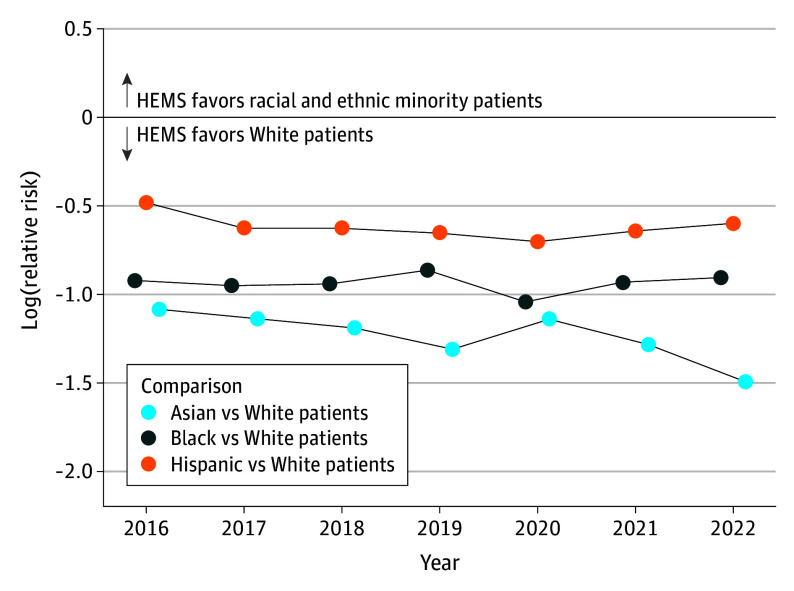
Temporal Trends in the Log-Relative Risk of Helicopter Emergency Medical Services (HEMS) A y-axis value less than 0 indicates lower helicopter use in the index group (Asian, Black, or Hispanic patients) relative to the referent group (White patients). We analyzed 356 576 patients (age >15 years) who sustained a severe injury and required an urgent surgical procedure or intensive care unit admission at a level I or II trauma center reporting to the National Trauma Data Bank from 2016 to 2022.

**Figure 3.**
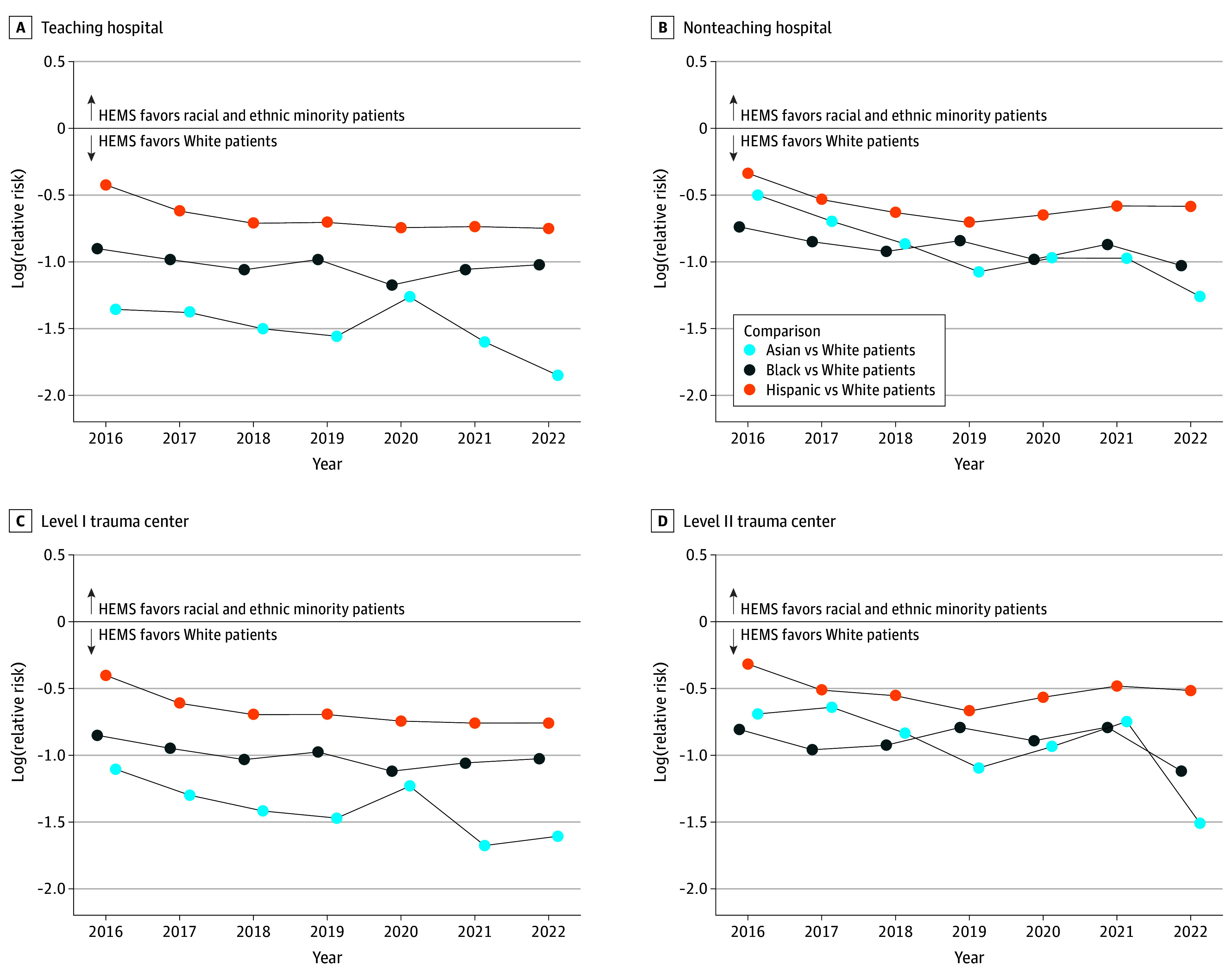
Temporal Trends in the Log-Relative Risk of Helicopter Emergency Medical Services (HEMS) A y-axis value less than 0 indicates lower HEMS use in the index group (Asian, Black, or Hispanic patients) relative to the referent group (White patients). We analyzed 356 576 patients (age >15 years) who sustained a severe injury and required an urgent surgical procedure or intensive care unit admission at a level I or II trauma center reporting to the National Trauma Data Bank from 2016 to 2022.

**Figure 4.**
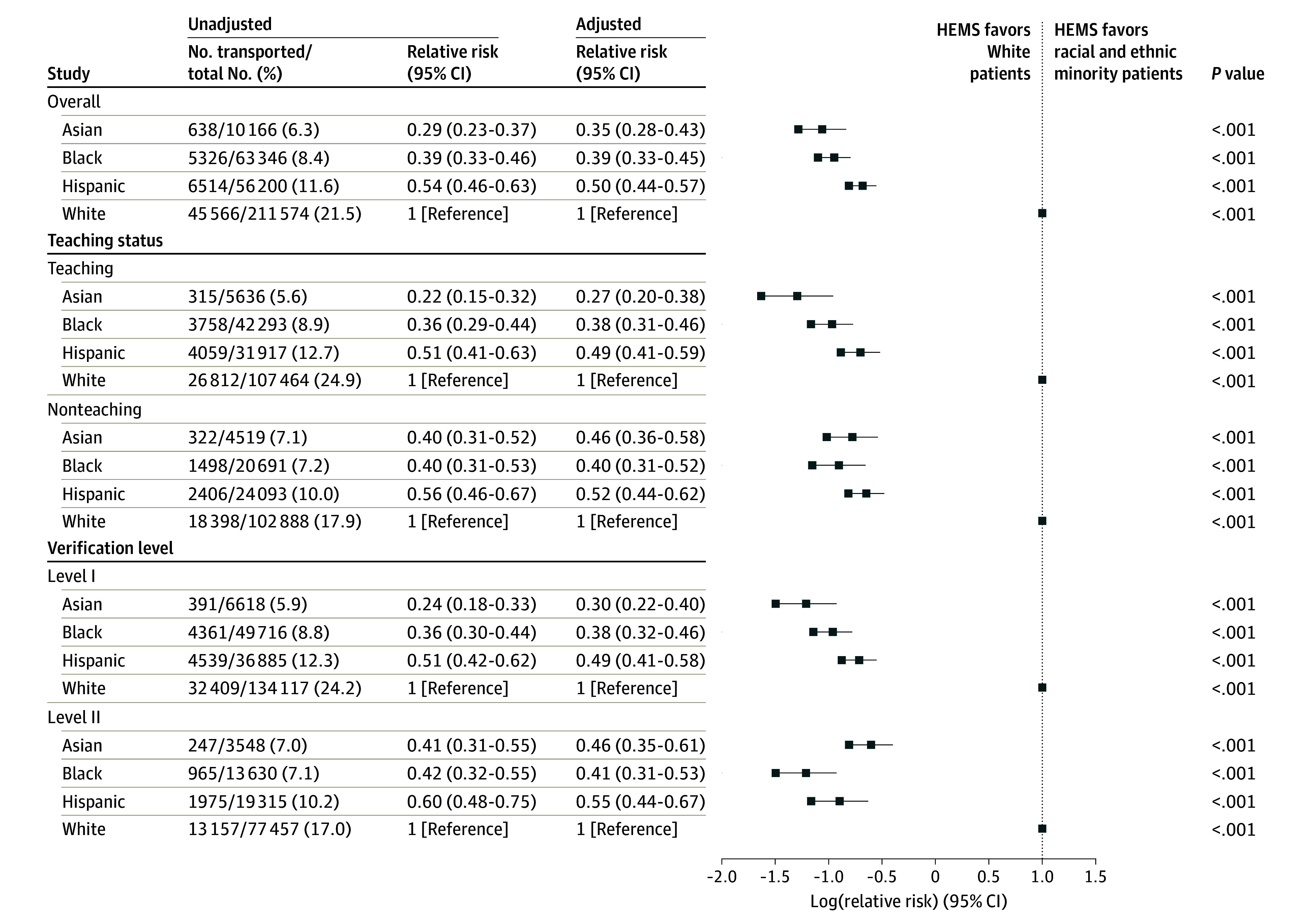
Unadjusted and Adjusted Relative Risk of Helicopter Emergency Medical Services (HEMS) Use Patients were older than 15 years and sustained severe trauma that required either urgent surgery or intensive care unit admission. Data are from the National Trauma Data Bank, 2016 to 2022.

After adjusting for patient and hospital characteristics, all racial and ethnic minority patients were consistently less likely to receive helicopter emergency medical services (HEMS) compared with White patients. Specifically, Asian patients were 42% less likely to receive helicopter transport compared with White patients (6.8% vs 21.8%, respectively; aRR, 0.38; 95% CI, 0.30-0.48; *P* < .001). In addition, Black patients were less likely to receive helicopter transport compared with White patients (8.7% vs 21.8%, respectively; aRR, 0.42; 95% CI, 0.36-0.49; *P* < .001). Similarly, Hispanic patients (11.2% vs 21.8% for White patients; aRR, 0.54; 95% CI, 0.48-0.61; *P* < .001) were also less likely to receive helicopter transport compared with White patients (Figure 2). We found no statistical evidence of the disparity narrowing during the study period (likelihood ratio test for the interaction between race and ethnicity category and year, *P* = .84) (Figure 3 and Figure 4).

### Racial and Ethnic Disparity by Hospital Characteristics and Insurance Status

We found statistical evidence that the disparity varied according to hospital characteristics (based on the likelihood ratio test for the interaction of race and ethnicity category with teaching status, verification level, and hospital control). The disparity between Asian and White patients was largest in teaching hospitals (6.0% vs 25.0%, respectively; aRR, 0.29; 95% CI, 0.21-0.40; *P* < .001) and level I trauma centers (6.5% vs 24.3%, respectively; aRR, 0.33; 95% CI, 0.24-0.44; *P* < .001). Similarly, the most substantial disparities in HEMS use between Black and White patients were noted in teaching hospitals (9.0% vs 25.0%, respectively; aRR, 0.41; 95% CI, 0.33-0.50; *P* < .001) and level I trauma centers (8.9% vs 24.3%, respectively; aRR, 0.40; 95% CI, 0.34-0.49; *P* < .001). The disparity between Hispanic and White patients was largest in teaching hospitals (13.3% vs 25.0%, respectively; aRR, 0.41; 95% CI, 0.33-0.50; *P* < .001).

### Sensitivity Analyses

Our findings remained consistent after repeating the analysis to adjust for an indicator of the distance between the injury site and the receiving hospital among a subset of the study population with information on transport times (eFigure 1 in Supplement 1). Repeating the analysis with 1:1 propensity score matching including a distance indicator among a subset of patients with information on transport times did not change our findings (eFigure 2 in Supplement 1). Stratified by insurance status, both privately and publicly insured patients displayed similar disparities in terms of HEMS use, with the effect being most pronounced in publicly insured Asian patients (eFigure 6 in Supplement 1). Repeating our analyses in strata according to injuries demonstrated confirmatory results regardless of mechanism or severity of injury (eFigures 5 and 7 in Supplement 1). We also observed consistent results within a subset of patients whose distance between accident site and hospital was greater than 15 miles. Moreover, the E-value analysis showed that our findings were robust to the potential influence of unmeasured confounders. The E-values ranged from 2.90 to 4.08, indicating that any unmeasured confounder would require a minimum association with both race and ethnicity and helicopter transport, with a relative risk of no less than 2.90, to reduce the confidence interval limits to 1.0.

## Discussion

In this population-based study, we examined the patterns of helicopter ambulance use across different racial and ethnic groups. Despite the clear survival benefits of helicopter transport in severe trauma situations, racial and ethnic minority populations, especially Asian and Black patients, were less likely to be airlifted compared with White patients. These disparities were most pronounced in patients treated at teaching hospitals and level I trauma centers, raising questions about the modifiable barriers preventing equitable access to helicopter ambulances in larger hospitals. In addition, our temporal analysis revealed that these disparities did not narrow over time, challenging the effectiveness of current efforts to expand helicopter ambulance programs. Our conservative methodical approach, which included several sensitivity analyses along with a rigorous propensity score matching, was designed to ensure a fair comparison among racial and ethnic groups.

Even against a backdrop of increasing awareness of health care disparities in the US, we found no evidence that the racial and ethnic gaps in use of helicopter transport were narrowing over time, indicating that the recent expansion of helicopter ambulances^9^ has not translated into equitable access for all racial and ethnic groups. This stagnation calls for a substantive shift from passive acknowledgment to active redressal of health care inequities. Recent academic contributions have emphasized the inadequacy of merely recognizing these disparities. Instead, we must address their underlying causes, which are rooted not just in socioeconomic conditions but also in a long history of racial discrimination and systemic barriers that persistently influence health care delivery.^22^

The persisting underrepresentation of racial and ethnic minorities in helicopter transport, despite its association with improved survival,^3,4,5,7,23,24^ signals a need for introspection within the trauma system to ensure that lifesaving interventions are neither advertently nor inadvertently influenced by race or ethnicity. We advocate for a multifaceted approach to narrow the observed racial and ethnic disparities in helicopter deployment. First, the role of EMS personnel in determining the optimal transport strategy cannot be overstated.^25,26^ However, the lack of structured protocols^26^ and ongoing professional education for EMS personnel, as noted in recent surveys,^27^ presents a significant barrier to optimizing transport strategies and mitigating disparities. As the evidence base and criteria for helicopter transport use evolve,^28^ EMS professionals may need to receive ongoing training that not only covers specific patient needs but also fosters an inclusive approach that recognizes the specific needs of diverse populations, encouraging EMS personnel to meet each patient where they are.

Another way to address these disparities is by promoting further reliance on triage scores based on evidence-based criteria to identify trauma patients who are most likely to benefit from helicopter transport. EMS personnel can use these tools at the time of the initial emergency call to improve dispatch accuracy and, by extension, patient outcomes.^29^ The Air Medical Prehospital Triage (AMPT) score, which was validated in recent studies,^30^ is a promising tool for improving triage and ensuring that helicopter transport decisions are driven by patient needs rather than nonclinical factors, such as race or ethnicity. In addition, the use of empirical triage scores will reduce the overtriaging of patients with minor injuries to helicopter transport.^31,32^ Thus, patients who are most likely to benefit from helicopter transport, including potentially underserved racial and ethnic minority patients, will be more likely to receive it.^32,33^

In addition to implementing structured triage systems (eg, using the AMPT score), structured communication between EMS personnel may help them make unbiased assessments of the need for helicopter transport.^34^ A recent consensus-building study proposed a model that addresses one potential barrier to equitable access to helicopter ambulance services: the variability and sometimes inadequacy of information exchange among dispatch centers, ground EMS teams, and helicopter EMS teams.^35^ Structured communication protocols could prevent nonclinical factors such as race and ethnicity from influencing the decision to dispatch helicopter ambulance services.

Finally, we need to continue improving the systematic reporting of major incidents with ongoing data collection and analysis to better understand and mitigate disparities in HEMS use, thereby improving future responses.^36,37^ By documenting and analyzing HEMS involvement in major incidents, emergency medical systems can identify gaps in service delivery and develop targeted interventions to ensure that all patients, regardless of their racial or ethnic background, have access to lifesaving aerial medical services.

The financial aspects of helicopter EMS are important considerations for understanding the broader implications of its use. Helicopter transport costs are typically substantial and can vary depending on the region, the organization, and the specifics of the transport. Payment for these services is generally covered by a combination of private insurance, government programs like Medicare and Medicaid, and out-of-pocket payments by patients.^38^ The proportion covered by insurance vs what patients may need to pay out of pocket can depend on the individual’s insurance plan, the medical necessity of the transport, and whether the organization is in network. Nonetheless, the financial argument should not negate the need for equitable utilization of lifesaving services such as HEMS.

### Limitations

Our study has some limitations that warrant consideration. First, while large datasets can make small differences statistically significant, our findings reveal substantial differences in helicopter transport. For example, the adjusted RR for Asian compared with White patients was 0.38, which suggests that White patients were 2.63 times more likely than Asian patients to receive helicopter transport. A similar magnitude was observed when comparing Black and White patients. These results remained consistent in sensitivity analyses, suggesting they are not solely due to the large sample size. Second, our categorization of racial and ethnic groups does not account for individuals with multiracial or multiethnic backgrounds, which may obscure the experiences and outcomes of these populations in our analysis. Third, despite using conservative statistical methods, unmeasured confounding remains a possibility; factors like socioeconomic status, geographic accessibility beyond distance, and specific hospital protocols may have influenced helicopter use and outcomes. Although we adjusted for within-hospital clustering, this may not fully address systemic issues, such as whether helicopters are more accessible in predominantly White areas. Additionally, the NTDB does not capture the decision-making process during 911 calls that influences EMS deployment, a critical factor in emergency transport. Future research could explore disparities in 911 call quality, as equitable information at this stage is vital for fair EMS deployment. Despite these challenges, the NTDB stands as one of the most extensive and reliable trauma registries in the US.

## Conclusions

In this population-based study, we found that racial and ethnic minority patients, particularly Black and Hispanic patients, and notably those received by larger hospitals, were disproportionately less likely to receive airlift services in comparison with White patients. This discrepancy persisted over time, bringing into question the effectiveness of ongoing efforts to broaden the scope of helicopter ambulance services in the US. We advocate for a multifaceted approach that includes enhancing the systematic reporting of major incidents, refining triage guidelines, and providing continuous training for EMS personnel.

## Notes

Supplement 1. **eFigure 1.** Sensitivity analysis by excluding injuries occurring <15 miles from receiving hospital **eFigure 2.** Sensitivity analyses showing the unadjusted and propensity score-adjusted relative risk of helicopter EMS utilization in patients (>15 years of age) who sustained severe trauma and required either urgent surgery or ICU admission **eFigure 3.** 1:1 Propensity score-adjusted relative risk of in hospital mortality in patients (>15 years of age) who sustained severe trauma and required either urgent surgery or ICU admission **eFigure 4.** Sensitivity analysis by adjusting for the estimated distance between injury site and receiving hospital, and excluding injuries occurring <15 miles from receiving hospital **eFigure 5.** Sensitivity analysis summarizing unadjusted and adjusted relative risk of helicopter EMS utilization in patients (>15 years of age) who sustained severe trauma and required either urgent surgery or ICU admission by injury type **eFigure 6.** Sensitivity analysis summarizing unadjusted and adjusted relative risk of helicopter EMS utilization in patients (>15 years of age) who sustained severe trauma and required either urgent surgery or ICU admission by insurance type **eFigure 7.** Sensitivity analysis summarizing unadjusted and adjusted relative risk of helicopter EMS utilization in patients (>15 years of age) who sustained severe trauma and required either urgent surgery or ICU admission by severity of injury **eMethods.** Additional details about the sensitivity analyses
eReferences

Supplement 2. Data sharing statement
