# Supplementary material for: Racial and Ethnic Disparities in Use of Helicopter Transport After Severe Trauma in the US
Source: JAMA Surg. 2025 Jan 22;160(3):313–21. doi: 10.1001/jamasurg.2024.6402 (PMC11904720; doi:10.1001/jamasurg.2024.6402)
Supplement: Supplement 1. — eFigure 1. Sensitivity analysis by excluding injuries occurring <15 miles from receiving hospital eFigure 2. Sensitivity analyses showing the unadjusted and propensity score-adjusted relative risk of helicopter EMS utilization in patients (>15 years of age) who sustained severe trauma and required either urgent surgery or ICU admission eFigure 3. 1:1 Propensity score-adjusted relative risk of in hospital mortality in patients (>15 years of age) who sustained severe trauma and required either urgent surgery or ICU admission eFigure 4. Sensitivity analysis by adjusting for the estimated distance between injury site and receiving hospital, and excluding injuries occurring <15 miles from receiving hospital eFigure 5. Sensitivity analysis summarizing unadjusted and adjusted relative risk of helicopter EMS utilization in patients (>15 years of age) who sustained severe trauma and required either urgent surgery or ICU admission by injury type eFigure 6. Sensitivity analysis summarizing unadjusted and adjusted relative risk of helicopter EMS utilization in patients (>15 years of age) who sustained severe trauma and required either urgent surgery or ICU admission by insurance type eFigure 7. Sensitivity analysis summarizing unadjusted and adjusted relative risk of helicopter EMS utilization in patients (>15 years of age) who sustained severe trauma and required either urgent surgery or ICU admission by severity of injury eMethods. Additional details about the sensitivity analyses eReferences [file jamasurg-e246402-s001.pdf]

Supplemental Online Content

Mpody C, Rudolph MI, Bastien A, et al. Racial and ethnic disparities in use of helicopter transport after severe trauma. *JAMA Surg*. Published online January 22, 2025. doi:10.1001/jamasurg.2024.6402

eFigure 1. Sensitivity analysis by excluding injuries occurring <15 miles from receiving hospital

eFigure 2. Sensitivity analyses showing the unadjusted and propensity score-adjusted relative risk of helicopter EMS utilization in patients (>15 years of age) who sustained severe trauma and required either urgent surgery or ICU admission

eFigure 3. 1:1 Propensity score-adjusted relative risk of in hospital mortality in patients (>15 years of age) who sustained severe trauma and required either urgent surgery or ICU admission

eFigure 4. Sensitivity analysis by adjusting for the estimated distance between injury site and receiving hospital, and excluding injuries occurring <15 miles from receiving hospital

eFigure 5. Sensitivity analysis summarizing unadjusted and adjusted relative risk of helicopter EMS utilization in patients (>15 years of age) who sustained severe trauma and required either urgent surgery or ICU admission by injury type

eFigure 6. Sensitivity analysis summarizing unadjusted and adjusted relative risk of helicopter EMS utilization in patients (>15 years of age) who sustained severe trauma and required either urgent surgery or ICU admission by insurance type

eFigure 7. Sensitivity analysis summarizing unadjusted and adjusted relative risk of helicopter EMS utilization in patients (>15 years of age) who sustained severe trauma and required either urgent surgery or ICU admission by severity of injury

eMethods. Additional details about the sensitivity analyses

eReferences

This supplemental material has been provided by the authors to give readers additional information about their work.

© 2025 Mpody C et al. *JAMA Surgery*.

	Unadjusted			Adjusted	
	HEMS transport/N (%)	Relative risk (95% CI)	*P*-value	Relative risk (95% CI)	*P*-value
**Part A: Overall**					
White	27896/127878(21.8)	Reference	--	Reference	--
Black	3004/34510(8.7)	0.40(0.33—0.48)	<0.01	0.42(0.36—0.49)	<0.01
Hispanic	4079/32959(12.4)	0.57(0.49—0.66)	<0.01	0.54(0.48—0.61)	<0.01
Asian	383/5601(6.8)	0.31(0.25—0.40)	<0.01	0.38(0.30—0.48)	<0.01
**Part B: By teaching status (LRT *P*-value for interaction with race or ethnicity < 0.01)**					
**Teaching**					
White	16110/64319(25.0)	Reference	--	Reference	--
Black	2161/23906(9.0)	0.36(0.29—0.45)	<0.01	0.41(0.33—0.50)	<0.01
Hispanic	2533/19047(13.3)	0.53(0.43—0.65)	<0.01	0.53(0.44—0.62)	<0.01
Asian	178/2982(6.0)	0.24(0.17—0.34)	<0.01	0.29(0.21—0.40)	<0.01
**Non-teaching**					
White	11538/62731(18.4)	Reference	--	Reference	--
Black	799/10368(7.7)	0.42(0.33—0.53)	<0.01	0.43(0.35—0.54)	<0.01
Hispanic	1511/13763(11.0)	0.60(0.49—0.72)	<0.01	0.57(0.48—0.68)	<0.01
Asian	204/2610(7.8)	0.42(0.31—0.57)	<0.01	0.51(0.38—0.68)	<0.01
**Part C: By verification level (LRT *P*-value for interaction with race or ethnicity < 0.01)**					
**Level I**					
White	19370/79646(24.3)	Reference	--	Reference	--
Black	2407/26988(8.9)	0.37(0.30—0.45)	<0.01	0.40(0.34—0.49)	<0.01
Hispanic	2783/21815(12.8)	0.52(0.43—0.63)	<0.01	0.52(0.44—0.60)	<0.01
Asian	229/3528(6.5)	0.27(0.2—0.36)	<0.01	0.33(0.24—0.44)	<0.01
**Level II**					
White	8526/48232(17.7)	Reference	--	Reference	--
Black	597/7522(7.9)	0.45(0.33—0.60)	<0.01	0.46(0.35—0.60)	<0.01
Hispanic	1296/11144(11.6)	0.66(0.53—0.81)	<0.01	0.61(0.50—0.74)	<0.01
Asian	154/2073(7.4)	0.42(0.29—0.61)	<0.01	0.49(0.35—0.70)	<0.01

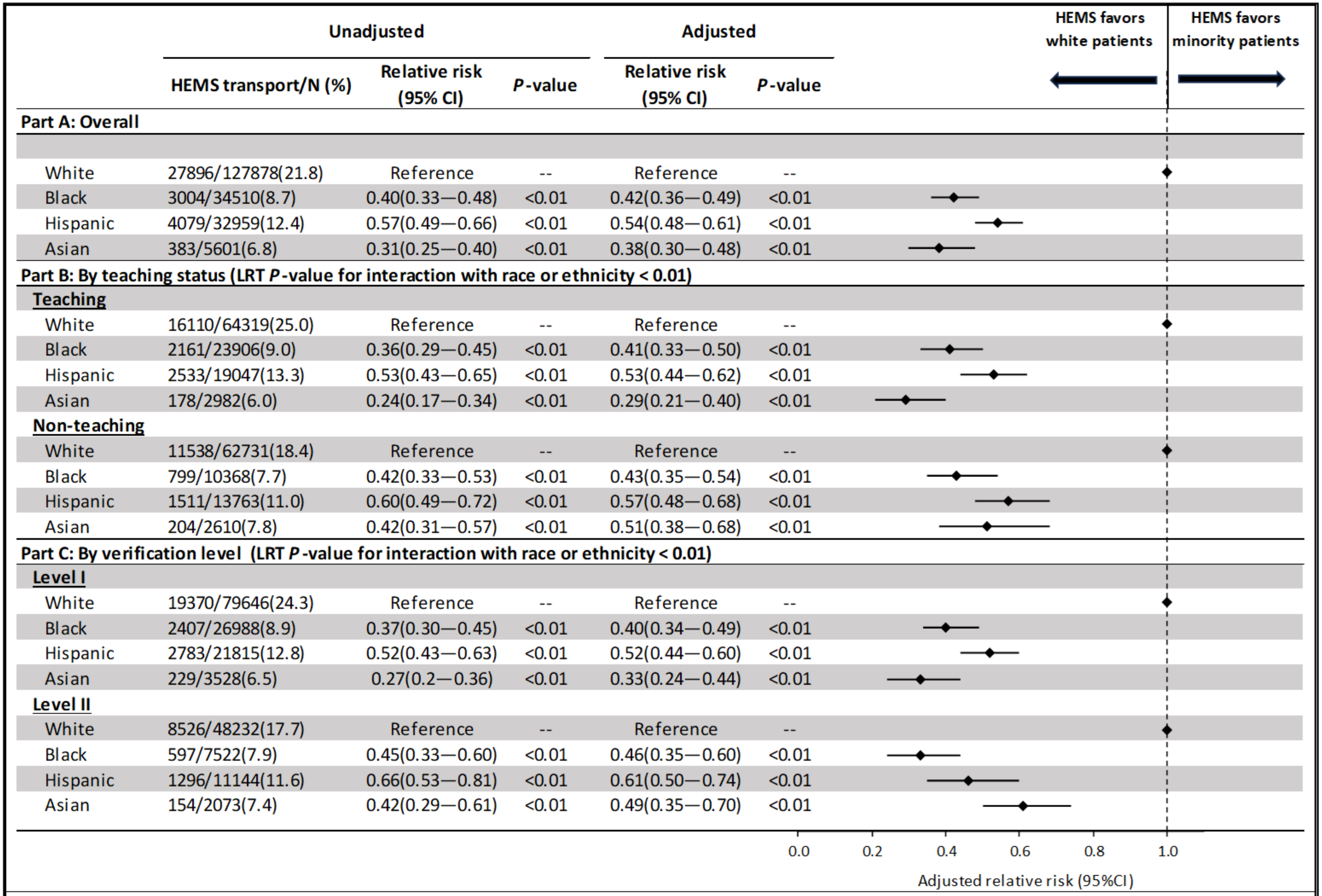

Figure S1. Sensitivity analysis by excluding injuries occuring <15 miles from receiving hospital. **Overall (Part A), by hospital teaching status (Part B), and by verification level (Part C)**. NTDB 2016–2022. Abbreviations: CI, confidence interval; NTDB, National Trauma Data Bank; HEMS, helicopter emergency medical service; EMS, emergency medical service. Among a subset (n=200,948) of patients with information on travel durations.

© 2025 Mpody C et al. *JAMA Surgery*.

	HEMS transport (%)	Propensity score adjusted relative risk (95% CI)	*P*-value
White	45566/211574(21.5)	Reference	--
Black	5326/63346(8.4)	0.40(0.34—0.46)	<0.01
Hispanic	6514/56200(11.6)	0.51(0.44—0.58)	<0.01
Asian	638/10166(6.3)	0.35(0.28—0.44)	<0.01

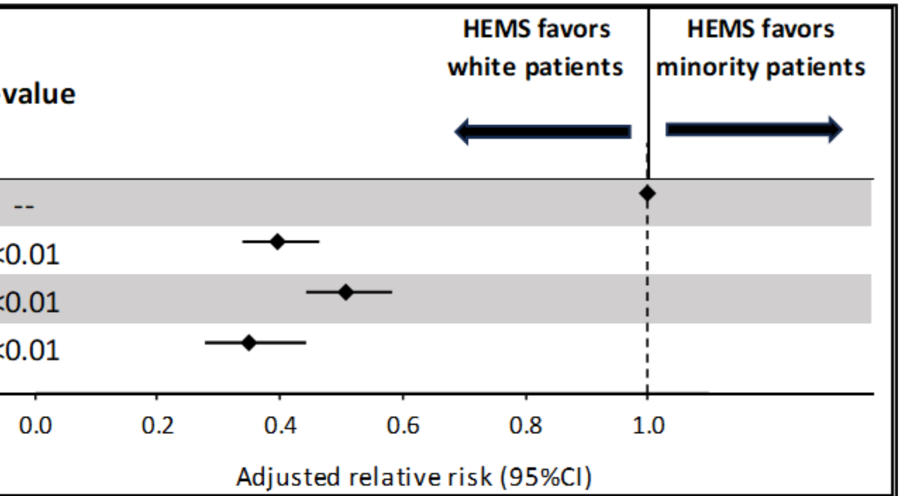

Figure S2. Sensitivity analyses showing the unadjusted and propensity score-adjusted relative risk of helicopter EMS utilization in patients (>15 years of age) who sustained severe trauma and required either urgent surgery or ICU admission. NTDB 2016–2022. Abbreviations: CI, confidence interval; NTDB, National Trauma Data Bank; HEMS, helicopter emergency medical service; EMS, emergency medical service.

© 2025 Mpody C et al. *JAMA Surgery*.

	Mortality (%)	Propensity-score adjusted relative risk (95% CI)	*P*-value
Ground ambulance	11458/26871(42.6)	Reference	--
Helicopter ambulance	10124/26871(37.7)	0.87(0.84—0.90)	<0.01

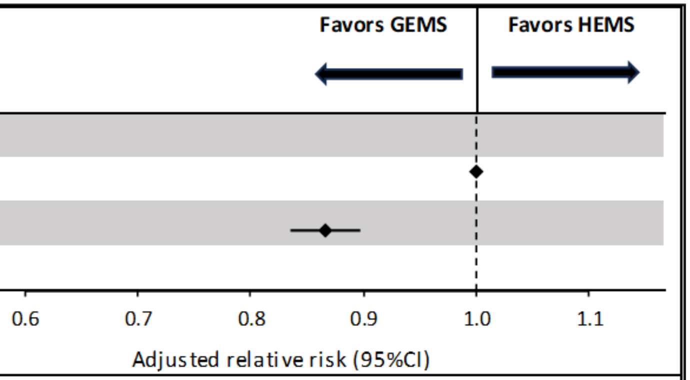

Figure S3. 1:1 Propensity score-adjusted relative risk of in hospital mortality in patients (>15 years of age) who sustained severe trauma and required either urgent surgery or ICU admission. NTDB 2016–2022. Abbreviations: CI, confidence interval; NTDB, National Trauma Data Bank; HEMS, helicopter emergency medical service; EMS, emergency medical service.

© 2025 Mpody C et al. *JAMA Surgery*.

	Unadjusted			Adjusted	
	HEMS transport/N (%)	Relative risk (95% CI)	*P*-value	Relative risk (95% CI)	*P*-value
**Blunt**					
White	27896/127878(21.8)	Reference	--	Reference	--
Black	3004/34510(8.7)	0.48(0.41—0.56)	<0.01	0.41(0.35—0.48)	<0.01
Hispanic	4079/32959(12.4)	0.58(0.50—0.66)	<0.01	0.51(0.45—0.58)	<0.01
Asian	383/5601(6.8)	0.29(0.23—0.37)	<0.01	0.34(0.27—0.42)	<0.01

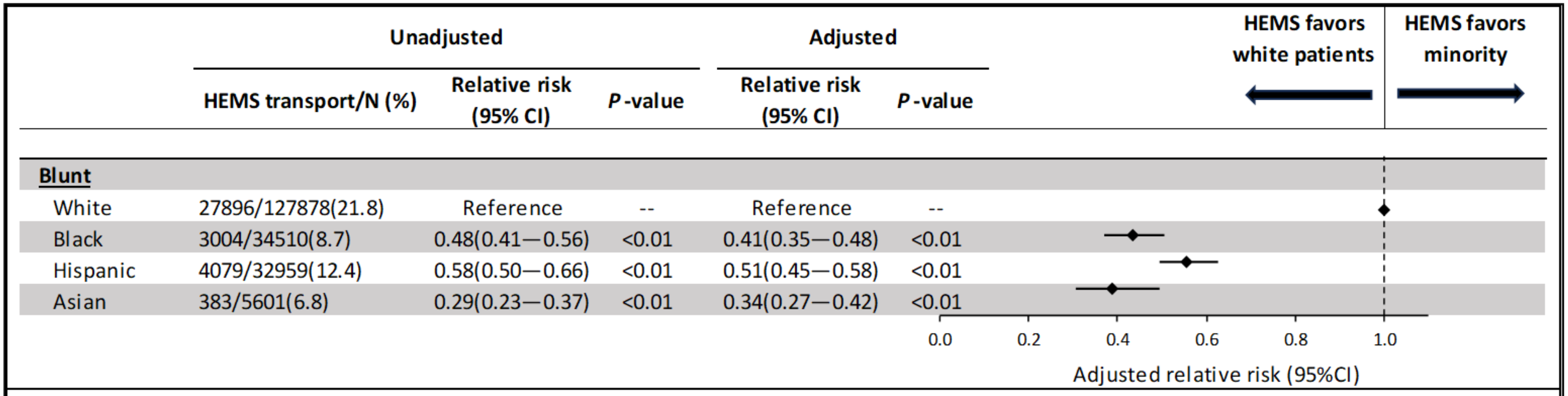

Figure S4. Sensitivity analysis by adjusting for the estimated distance between injury site and receiving hospital, and excluding injuries occuring <15 miles from receiving hospital. NTDB 2016–2022. Abbreviations: CI, confidence interval; NTDB, National Trauma Data Bank; HEMS, helicopter emergency medical service; EMS, emergency medical service. Among a subset (n=200,948) of patients with information on travel durations.

© 2025 Mpody C et al. *JAMA Surgery*.

	Unadjusted			Adjusted	
	HEMS transport/N (%)	Relative risk (95% CI)	*P* -value	Relative risk (95% CI)	*P* -value
**Blunt**					
White	41165/191331(21.5)	Reference	--	Reference	--
Black	4165/40438(10.3)	0.48(0.41—0.56)	<0.01	0.41(0.35—0.48)	<0.01
Hispanic	5666/45705(12.4)	0.58(0.50—0.66)	<0.01	0.51(0.45—0.58)	<0.01
Asian	583/9220(6.3)	0.29(0.23—0.37)	<0.01	0.34(0.27—0.42)	<0.01
**Penetrating**					
White	2824/13663(20.7)	Reference	--	Reference	--
Black	985/21025(4.7)	0.23(0.18—0.28)	<0.01	0.28(0.23—0.34)	<0.01
Hispanic	502/8169(6.1)	0.30(0.23—0.38)	<0.01	0.35(0.28—0.43)	<0.01
Asian	28/544(5.1)	0.25(0.15—0.42)	<0.01	0.28(0.16—0.49)	<0.01

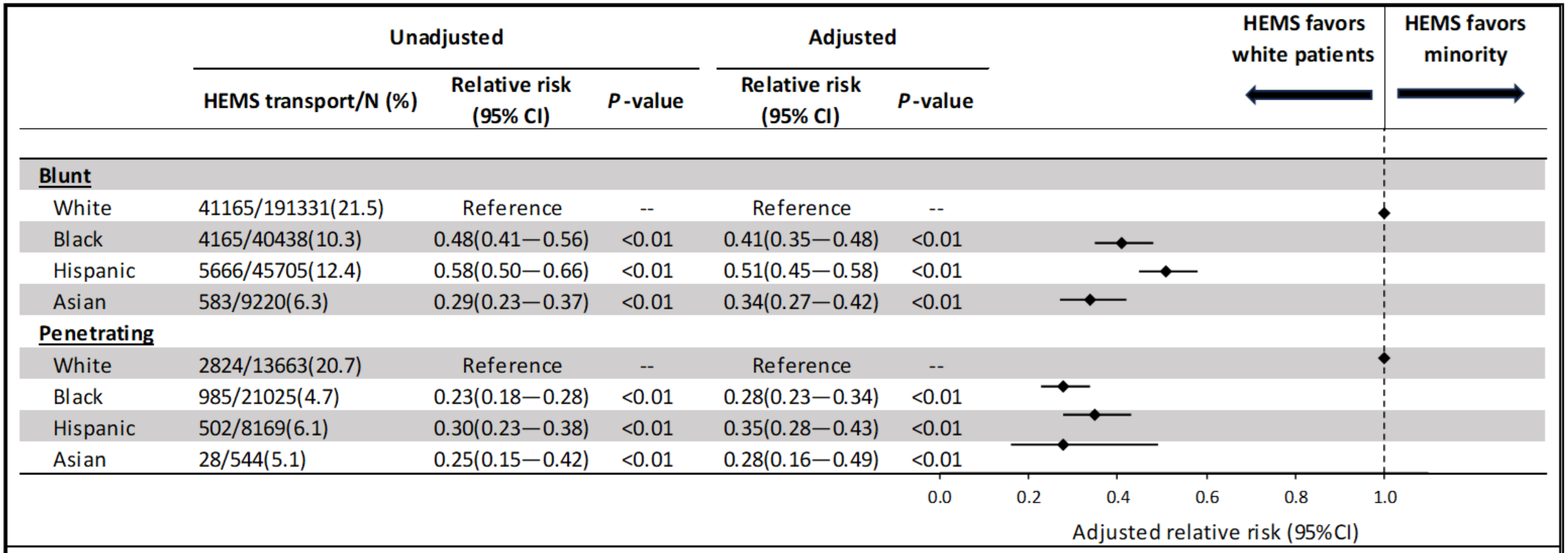

Figure S5. Sensitivity analysis summarizing unadjusted and adjusted relative risk of helicopter EMS utilization in patients (>15 years of age) who sustained severe trauma and required either urgent surgery or ICU admission. **By injury type**. NTDB 2016–2022. Abbreviations: CI, confidence interval; NTDB, National Trauma Data Bank; HEMS, helicopter emergency medical service; EMS, emergency medical service.

© 2025 Mpody C et al. *JAMA Surgery*.

	Unadjusted			Adjusted	
	HEMS transport/N (%)	Relative risk (95% CI)	*P*-value	Relative risk (95% CI)	*P*-value
**Private insurance**					
White	27906/109530(25.5)	Reference	--	Reference	--
Black	2965/31093(9.5)	0.37(0.31—0.45)	<0.01	0.39(0.33—0.47)	<0.01
Hispanic	3915/29436(13.3)	0.52(0.46—0.60)	<0.01	0.53(0.48—0.59)	<0.01
Asian	442/4937(9)	0.35(0.29—0.43)	<0.01	0.41(0.33—0.50)	<0.01
**Public insurance**					
White	15462/93383(16.6)	Reference	--	Reference	--
Black	1931/28699(6.7)	0.41(0.34—0.49)	<0.01	0.37(0.31—0.43)	<0.01
Hispanic	2155/24087(8.9)	0.54(0.44—0.66)	<0.01	0.44(0.37—0.53)	<0.01
Asian	165/4840(3.4)	0.21(0.15—0.28)	<0.01	0.24(0.18—0.34)	<0.01

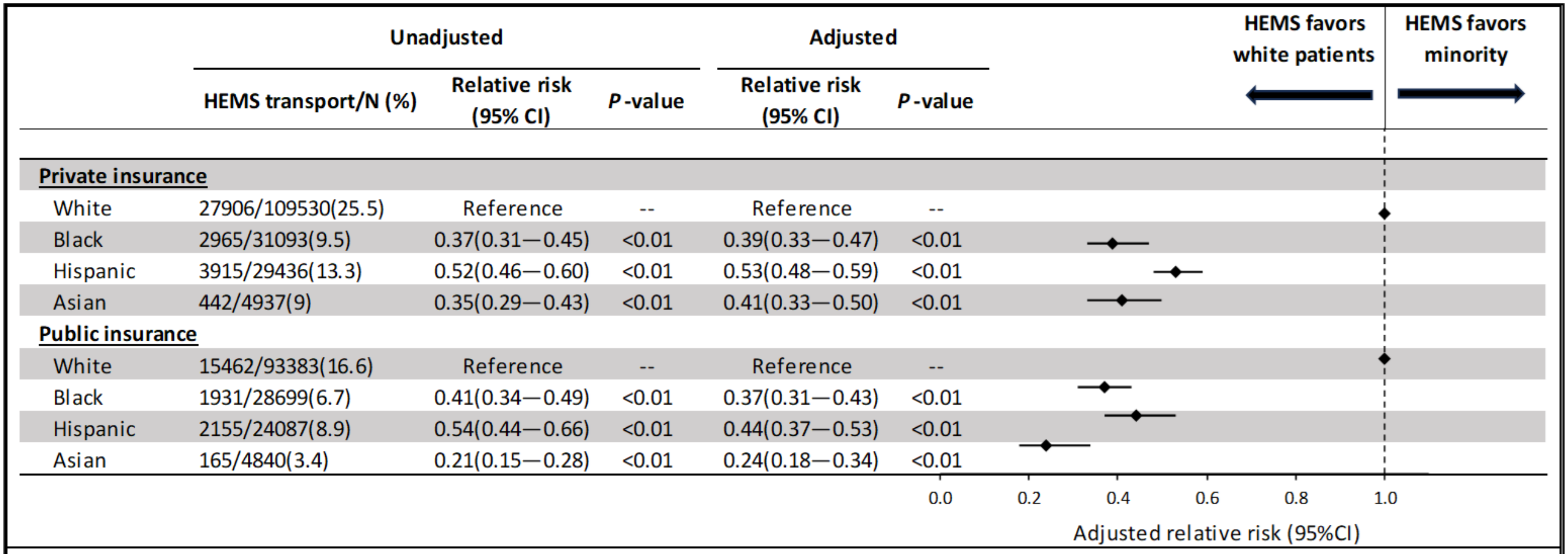

Figure S6. Sensitivity analysis summarizing unadjusted and adjusted relative risk of helicopter EMS utilization in patients (>15 years of age) who sustained severe trauma and required either urgent surgery or ICU admission. **By insurance type**. NTDB 2016–2022. Abbreviations: CI, confidence interval; NTDB, National Trauma Data Bank; HEMS, helicopter emergency medical service; EMS, emergency medical service.

© 2025 Mpody C et al. *JAMA Surgery*.

	Unadjusted			Adjusted	
	HEMS transport/N (%)	Relative risk (95% CI)	*P*-value	Relative risk (95% CI)	*P*-value
**Severe (ISS 16-24)**					
White	22429/112474(19.9)	Reference	--	Reference	--
Black	2505/31488(8.0)	0.40(0.34—0.47)	<0.01	0.39(0.33—0.45)	<0.01
Hispanic	3169/28736(11.0)	0.55(0.48—0.64)	<0.01	0.51(0.45—0.58)	<0.01
Asian	330/5157(6.4)	0.32(0.25—0.41)	<0.01	0.36(0.29—0.45)	<0.01
**Very severe (ISS ≥ 25)**					
White	23113/98839(23.4)	Reference	--	Reference	--
Black	2814/31760(8.9)	0.38(0.32—0.45)	<0.01	0.38(0.33—0.45)	<0.01
Hispanic	3344/27362(12.2)	0.52(0.44—0.62)	<0.01	0.49(0.43—0.57)	<0.01
Asian	308/4984(6.2)	0.26(0.20—0.34)	<0.01	0.31(0.24—0.40)	<0.01

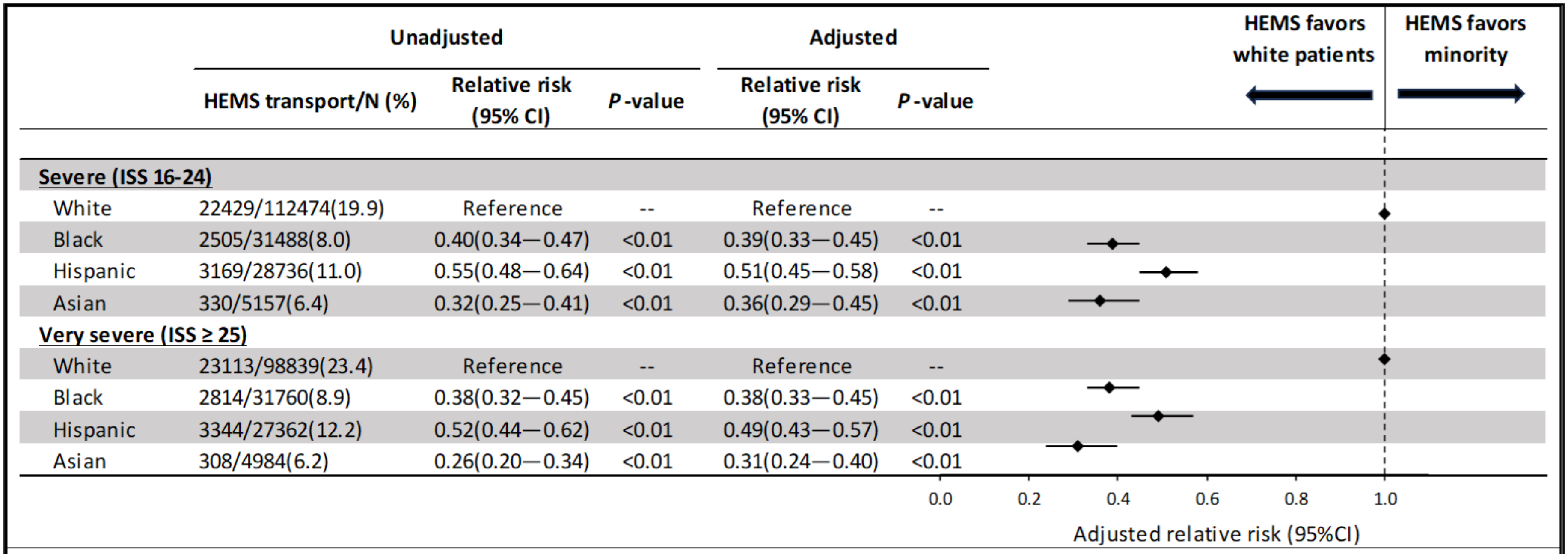

Figure S7. Sensitivity analysis summarizing unadjusted and adjusted relative risk of helicopter EMS utilization in patients (>15 years of age) who sustained severe trauma and required either urgent surgery or ICU admission. **By severity of injury**. NTDB 2016–2022. Abbreviations: CI, confidence interval; NTDB, National Trauma Data Bank; HEMS, helicopter emergency medical service; EMS, emergency medical service.

© 2025 Mpody C et al. *JAMA Surgery*.

eMethods. Additional details about the sensitivity analyses

For the propensity score matching, we selected patients with the highest empirical proportion of helicopter ambulance use as the reference group—in this case, White patients. Patients from each index group were matched 1:1 to patients from the referent group using propensity score matching without replacement. We applied the same method to create three propensity score–matched cohorts: Black vs. White, Hispanic vs. White, and Asian vs. White. We calculated the propensity scores using multivariable logistic regression. Post matching, covariate-specific imbalances were evaluated, with an absolute standard difference of ≥10% defining imbalance.[1,2]

The E-value measures the magnitude of association that an unmeasured confounder would need to have with both the exposure and outcome to nullify the observed associations, specifically to reduce the confidence interval limits to 1.0.[3] A larger E-value means that the unmeasured confounding would need to be larger to explain away the observed association.[3]

eReferences

1. Austin PC. Balance diagnostics for comparing the distribution of baseline covariates between treatment groups in propensity-score matched samples. *Stat Med*. Nov 10 2009;28(25):3083-107. doi:10.1002/sim.3697
2. Austin PC, Stuart EA. Moving towards best practice when using inverse probability of treatment weighting (IPTW) using the propensity score to estimate causal treatment effects in observational studies. *Stat Med*. Dec 10 2015;34(28):3661-79. doi:10.1002/sim.6607
3. VanderWeele TJ, Ding P. Sensitivity Analysis in Observational Research: Introducing the E-Value. *Annals of internal medicine*. Aug 15 2017;167(4):268-274. doi:10.7326/m16-2607

© 2025 Mpody C et al. *JAMA Surgery*.
