# Supplementary material for: Racial and Ethnic Disparities in Use of Helicopter Transport After Severe Trauma in the US
Source: JAMA Surg. 2025 Jan 22;160(3):313–21. doi: 10.1001/jamasurg.2024.6402 (PMC11904720; doi:10.1001/jamasurg.2024.6402)
Supplement: Supplement 2. — Data sharing statement [file jamasurg-e246402-s002.pdf]

Data Sharing Statement

Mpody. Racial and Ethnic Disparities in Use of Helicopter Transport After Severe Trauma in the US. *JAMA Surg*. Published January 22, 2025. doi:10.1001/jamasurg.2024.6402

Data
Data available: No

Additional Information
Explanation for why data not available: Available upon request
